# The Role of [^18^F]FDG PET Imaging for the Assessment of Vegetative State or Unresponsive Wakefulness Syndrome: A Systematic Review

**DOI:** 10.3390/diagnostics15111406

**Published:** 2025-05-31

**Authors:** Francesco Dondi, Nicola Latronico, Pietro Bellini, Silvia Lucchini, Luca Camoni, Michela Cossandi, Gian Luca Viganò, Giulia Santo, Francesco Bertagna

**Affiliations:** 1 Nuclear Medicine, Università degli Studi di Brescia, 25123 Brescia, Italy; francesco.bertagna@unibs.it; 2 Nuclear Medicine, ASST Spedali Civili di Brescia, 25123 Brescia, Italy; pietro.bellini@asst-spedalicivili.it (P.B.); silvia.lucchini@asst-spedalicivili.it (S.L.); michela.cossandi@asst-spedalicivili.it (M.C.); 3 Department of Anesthesia, Critical Care and Emergency, ASST Spedali Civili University Hospital, 25123 Brescia, Italy; nicola.latronico@unibs.it; 4 ASST Spedali Civili di Brescia, UOC Ingegneria Clinica, 25123 Brescia, Italy; 5 Department of Experimental and Clinical Medicine, “Magna Graecia” University of Catanzaro, 88100 Catanzaro, Italy; giulia.santo@unicz.it

**Keywords:** PET, PET/CT, ^18^F-fluorodeoxyglucose, positron emission tomography, vegetative state, unresponsive wakefulness syndrome, UWS, VS, disorder of consciousness, DOC

## Abstract

**Background**: Different evidence on the ability of [^18^F] fluorodeoxyglucose ([^18^F]FDG) positron emission tomography (PET) imaging to assess patients in the vegetative state (VS) or unresponsive wakefulness syndrome (UWS) has been reported. Therefore, this systematic review aimed to synthesize the existing literature on this topic. **Methods**: A wide literature search of the PubMed/MEDLINE, Scopus, and Embase databases was conducted to find relevant published articles investigating the role of [^18^F]FDG PET imaging in the assessment of VS/UWS. **Results**: Thirty-seven studies were included in the review, and the main fields of application of this imaging modality in these patients were the evaluation of hypometabolic patterns, differentiation of disorders of consciousness (DOC), prognostic value, and ability to assess the response to particular stimuli. **Conclusions**: The possible role of [^18^F]FDG PET imaging in the assessment of VS/UWS has emerged, in particular in the differential diagnosis of other DOC or prognosis. Some insights into its value in stimulation response and therapy evaluation have also been proposed. Standardization of protocols and larger prospective studies are needed to strengthen these clinical recommendations.

## 1. Introduction

Disorders of consciousness (DOC) encompass a variety of conditions, such as coma, minimally conscious state (MCS), vegetative state (VS), or unresponsive wakefulness syndrome (UWS) [1,2]. When VS lasts for more than 4 weeks, it is defined as persistent VS (PVS) [3].

VS/UWS is characterized by a state of wakefulness without awareness of self and the environment. Diagnosis is based on clinical assessment and is burdened with a high error rate since consciousness can be present in 5% to 15% of patients fulfilling the clinical criteria for VS/UWS [4]. Under these conditions, patients can open their eyes spontaneously, and electroencephalogram (EEG) testing can reveal the presence of sleep-wake cycles. Additionally, reflex behaviors are still present in these patients, and in particular, arousal by external stimulation or provocation may be present [1,5,6]. From a neurological and functional point of view, the presence of wakefulness suggests the preservation of brainstem function, whereas lack of awareness indicates an underlying cortical dysfunction [1].

The extent of structural brain damage causing VS/UWS can be assessed using different neuroimaging techniques, such as computed tomography (CT) and magnetic resonance imaging (MRI) [7]. Moreover, nuclear medicine techniques are used to determine the level of functional cerebral impairment in patients with DOC [8]. In particular, [18F]fluorodeoxyglucose ([^18^F]FDG) positron emission tomography (PET) imaging can evaluate the glycolytic activity of different structures and is useful for assessing different pathological conditions that affect the brain, such as neurodegenerative disorders or encephalitis [9,10,11,12,13,14]. The diagnostic value of PET imaging in the assessment of different conditions affecting the central nervous system (CNS) is also underlined by the fact that a high number of tracers, different from [^18^F]FDG, have also been studied and used to assess diverse pathologies that can involve these structures [15,16,17,18]. Several papers assessing the role of [^18^F]FDG for the assessment of different DOC have been published in recent years; however, a clear analysis of its added value and a comparison between them are not available, in particular in the case of VS/UWS.

The aim of this systematic review is, therefore, to summarize the available literature and define the role of [^18^F]FDG PET imaging in supporting a clinically defined diagnosis of VS/UWS.

## 2. Materials and Methods

This systematic review was performed according to the “Preferred Reporting Items for a Systematic Review and Meta-Analysis” (PRISMA 2020 statement), which was employed as a guide in its development. The complete PRSIMA checklist is available in the Supplementary Materials.

### 2.1. Search Strategy

A comprehensive search was conducted across three databases (PubMed/MEDLINE, Scopus, and Embase) to identify published articles addressing the role of [^18^F]FDG PET imaging in the assessment of VS or UWS. The algorithm used for the research was: “(“PET” OR “PET/CT” OR “PET/MRI” OR “positron emission tomography”) AND (“vegetative state” OR “unresponsive wakefulness syndrome”).

The search had no beginning date limit and was updated until 01 March 2025. Only articles published in English were considered. Preclinical studies, conference proceedings, reviews, editorials, and case reports were excluded. If two papers focused on the same cohort to perform research with the same aim, the more updated article was included in the review, while the other was excluded. The references of the retrieved articles were also screened for additional papers to expand the final database.

### 2.2. Study Selection

Two researchers (F.D. and F.B.) independently screened the titles and abstracts of the retrieved articles. The same two researchers independently reviewed the full-text versions of the identified articles to determine their eligibility for inclusion. The main question of the review was “What is the diagnostic role of [^18^F]FDG PET imaging in patients with VS/UWS? The eligibility criteria were chosen taking into account this question. Clinical studies reporting diagnostic information on the use of PET in the assessment of VS/UWS were deemed eligible for inclusion in this systematic review. Moreover, studies without a clear focus on VS/UWS subjects and those whose analyses were not specifically performed on these patients were excluded. Furthermore, papers with incomplete data, such as the precise number of patients in VS/UWS, were not considered for inclusion.

### 2.3. Quality Assessment

The quality assessment of these studies, including the risk of bias and applicability concerns, was carried out using the Quality Assessment of Diagnostic Accuracy Studies version 2 (QUADAS-2) evaluation [19]. Quality assessment was performed independently by the two reviewers, and discrepancies were resolved by consensus.

### 2.4. Data Extraction

The same two reviewers independently evaluated the retrieved studies to collect relevant information for the review. For each study included in the review, data concerning the basic information of the study, such as the first author’s name, year of publication, country of origin, design of the study, radiopharmaceutical used, and number of patients, were collected. Furthermore, information about the type of PET tomograph used, the activity of the injected tracer, and the main results were also collected. The main findings of the articles included in this review are therefore reported in the Results section. This study was conducted in accordance with the PRISMA guidelines.

## 3. Results

### 3.1. Literature Search

**Figure 1:**
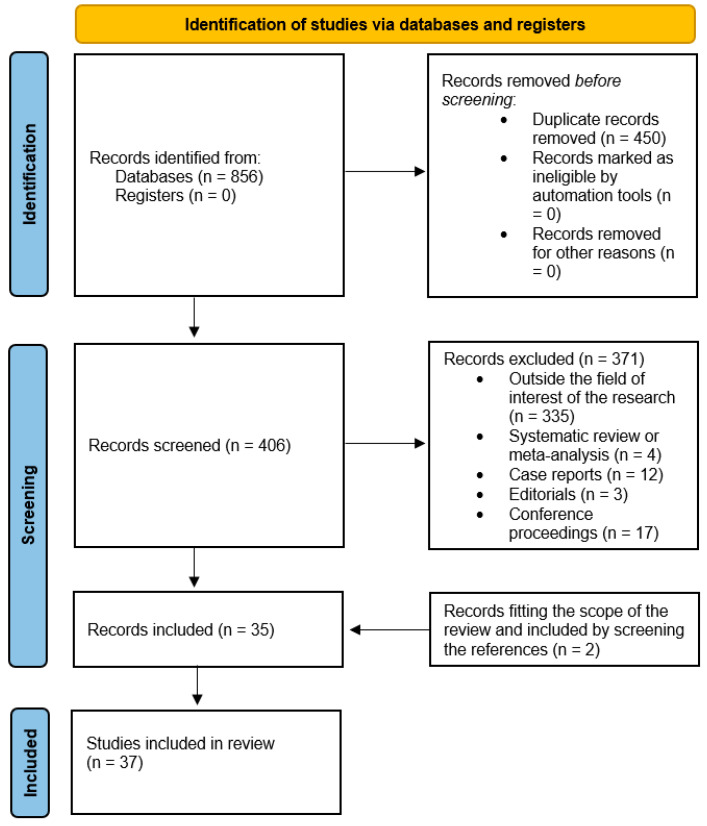
Flowchart of the research of eligible studies evaluating the role of [^18^F]FDG PET imaging in the assessment of VS/UWS.

The literature search retrieved 406 articles. After reviewing the titles and abstracts, 371 studies were excluded by applying the exclusion criteria mentioned As a consequence, 35 studies addressing the role of [^18^F]FDG PET imaging in the evaluation of VS/UWS were selected and retrieved in the full-text version [20,21,22,23,24,25,26,27,28,29,30,31,32,33,34,35,36,37,38,39,40,41,42,43,44,45,46,47,48,49,50,51,52,53,54]. Two additional studies were found after analyzing the reference lists of these articles [55,56]. Therefore, 37 studies were included in the review (Figure 1).

**Figure 2:**
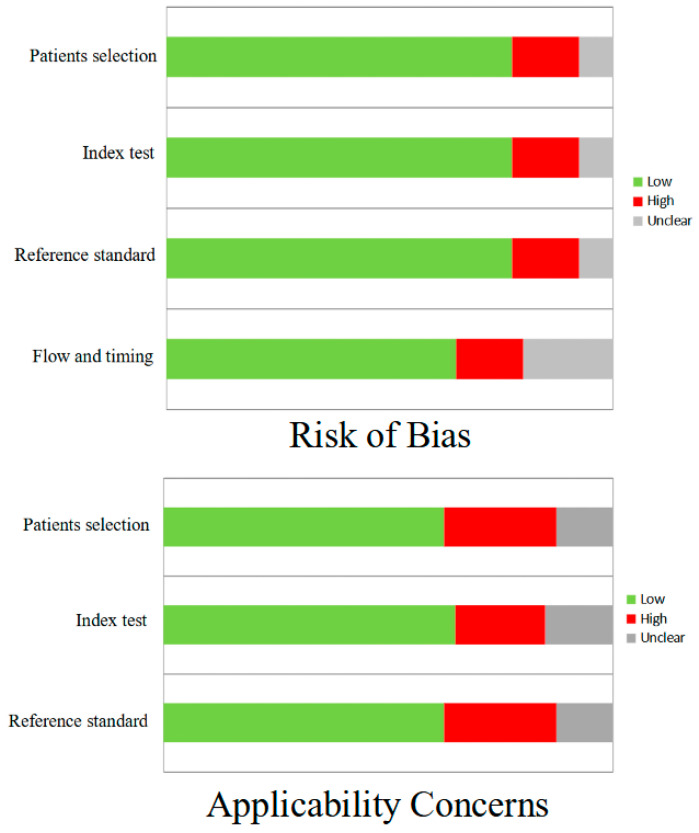
QUADAS-2 quality assessment for risk of bias and applicability concerns for the studies included in the review.

In general, the quality assessment using QUADAS-2 evaluation underlined the presence of a low risk of bias in most domains for all studies included in the review. The diagnosis of VS/UWS was performed in all cases according to current recommendations (or currently proposed criteria). Considering applicability concerns, a slightly higher risk of bias was observed in all domains (Figure 2). In particular, high technical variability derived from different PET protocols used in different studies was present; in some cases, the protocol was not clearly specified. Moreover, some methodological flaws were related to the fact that in some papers, small and mixed cohorts were considered, composed of different DOCs, resulting in a low number of VS/UWS patients examined with PET. Furthermore, non-consecutive enrollment and the fact that, by definition, the clinical assessment of these patients has the intrinsic limit of a subjective evaluation were issues regarding patient selection and reference standards.

Among the total number of studies included in the systematic review, 11 were retrospective [31,34,37,38,40,44,45,47,51,54,55], whereas 26 had a prospective design [20,21,22,23,24,25,26,27,28,29,30,32,33,35,36,39,41,42,43,46,48,49,50,52,53,56]. In terms of radiopharmaceuticals used, 32 studies were performed using [^18^F]FDG [21,22,23,27,39,40,41,42,43,44,45,46,47,48,49,50,51,52,53,54,56], 3 with both [^18^F]FDG and [^15^O]H_2_O [24,25,28], 1 with [^18^F]FDG and [^11^C]flumazenil [26] and 1 with [^15^O]CO_2_ and [^18^F]FDG [20]. Moreover, 10 studies were performed using a PET/computed tomography (CT) tomograph [33,37,39,40,42,46,50,52,53,54], 25 studies used PET tomographs [20,21,22,23,24,25,26,27,28,29,30,31,32,33,34,35,36,43,44,45,47,48,49,51,55,56], in one case the type of tomograph was not specified [38], and in one case a PET/MR tomograph was used [41]. The main characteristics of the studies and their results are briefly presented in Table 1 and Table 2.

### 3.2. The Role of [^18^F]FDG PET Imaging for the Evaluation of VS/UWS

As mentioned before, several papers have evaluated the ability of [^18^F]FDG PET imaging to assess brain metabolism in patients with VS/UWS. First, De Volder et al. [21] reported that the mean gray matter glucose utilization rate was lower in postanoxic patients than in healthy controls (HC), and that metabolism was significantly decreased mainly in VS/UWS patients compared to subjects who regained consciousness, and that VS/UWS and conscious postanoxic subjects had significantly different metabolic rates. Reduced metabolic activity was demonstrated in particular in the parieto-occipital cortex, but the frontal and frontomesial areas were also involved. Interestingly, visual cortex and striatal hypometabolism were present in patients with suspected blindness based on altered visual evoked potential.

Similarly, Tommasino et al. [22] revealed that mean glucose metabolism was significantly reduced in patients with coma, VS/UWS, or PVS compared to HC. PVS subjects had the lowest metabolic rate, while VS/UWS and coma subjects had almost no differences in regional metabolic activity, with the only exception of a higher metabolism in the primary visual cortex in VS/UWS patients.

Similar results were also proposed by Rudolf et al. [23], who assessed the role of [^18^F]FDG PET imaging in differentiating between acute VS and PVS and revealed that glucose metabolism was, in general, symmetrically reduced in all patients in their cohort. Moreover, compared to HC, metabolism was significantly decreased in all regions in VS/UWS patients, except for the brainstem. In addition, metabolic activity was slightly higher in patients who remained in VS/UWS than in those who died during the observation period of 12 months, but the difference was not statistically significant. With the exception of the frontal lobe, global and regional metabolic rates were higher in patients with acute VS than in patients with PVS.

Laurey et al. [55] evaluated patients in VS/UWS with [^18^F]FDG PET, reporting that they had significantly impaired glucose metabolism in the left and right middle and superior frontal gyri, the left inferior frontal gyrus, the left inferior parietal lobule, the left middle temporal gyrus, the right superior temporal gyrus, the posterior cingulate cortex/precuneus, and the left pre- and postcentral gyri. Moreover, compared to HC, a significant difference in effective connectivity between the left prefrontal and premotor cortices and the posterior cingulate cortex was demonstrated.

Beuthien-Baumann et al. [27] reported that patients in PVS demonstrated a reduction of both glucose metabolism and perfusion assessed with [^99m^Tc]hexamethylpropylene-amine-oxym ([^99m^Tc]HMPAO) single photon emission computed tomography (SPECT). The areas mainly involved were the frontal cortex and temporal cortex, and less frequently, the parietal cortex, occipital cortex, and cerebellum. In contrast, a relatively pronounced glucose uptake was reported in the vermis cerebelli in different subjects. Significant differences in general brain glucose uptake were reported for patients with PVS compared to HC, again with the exception of the vermis cerebelli. After normalization, PVS had higher relative mean values of glucose uptake in the left cerebellum and vermis cerebelli than normal subjects.

Coleman et al. [29] investigated the integrity of the homoeostatic coupling relationship between neuronal electrical function and cerebral metabolism in VS/UWS and MCS, reporting that the regional cerebral metabolic rate was not significantly different between the two conditions. Interestingly, a correlation between the ratio of slow to fast wave EEG activity and glucose metabolism was present in patients with MCS, while in VS/UWS subjects, the correlation was not confirmed.

Juengling et al. [30] aimed to separate functional and structural cerebral damage in PVS by the use of [^18^F]FDG PET and MRI. PET images showed a widespread pattern of hypometabolic areas in the parietal and frontotemporal cortices, cuneus/precuneus, cingulum, frontal medial and precentral gyrus, transverse temporal gyrus, and bilateral thalamus (mainly the dorsomedial subnucleus). Similar to voxel-based morphometry, all changes were symmetrical, and the overlap between significant clusters of each imaging modality was relatively small, including the right operculum and left medial temporal gyrus, right caudate, and parts of the cingulum.

More recently, Nakayama et al. [31] evaluated the relationship between regional cerebral glucose metabolism and consciousness disturbance in traumatic diffuse brain injuries. Compared to HC, patients (a cohort including higher brain dysfunction, MCS, and VS/UWS) showed marked bilateral hypometabolism in the medial prefrontal region, medial frontobasal region, anterior and posterior regions of the cingulate gyrus, and thalamus. Compared to those with MCS, patients in VS/UWS showed more widespread and prominent hypometabolism, especially in the medial prefrontal and medial frontobasal regions. Additionally, compared to HC, VS/UWS patients had a metabolic reduction in the thalamus, cingulate gyrus, cerebral peduncle, hypothalamus, subcallosal gyrus, inferior frontal gyrus, and medial frontal gyrus.

Bruno et al. [56] used [^18^F]FDG PET to document the neural correlate of ambiguous behavioral signs of consciousness in challenging VS/UWS patients. The authors reported that, compared to HC, patients with VS/UWS without visual fixation showed metabolic dysfunction in a widespread cerebral network encompassing the bilateral thalami and fronto-temporo-parietal associative cortices. The relatively spared areas were confined to the brainstem and cerebellum. Patients with visual fixation but with clinical characteristics typical of VS/UWS showed a similar pattern of metabolic dysfunction, and the direct comparison between these patients showed no significant differences. Therefore, the authors suggested that sustained visual fixation does not necessarily reflect consciousness and higher-order cortical brain function.

Kim et al. [32] investigated the changes in cerebral glucose metabolism in patients with PVS after acquired brain injury, reporting that compared to HC, they demonstrated a significantly decreased metabolism in the left precuneus, the posterior cingulate cortices and the left superior parietal lobule. In contrast, the cerebellar and right supramarginal cortex showed statistically increased metabolism in patients with PVS. In addition, considering all the cohorts, a decreased level of consciousness was significantly correlated with decreased regional cerebral glucose metabolism in both posterior cingulate cortices in patients with PVS.

An interesting research performed by Snyman et al. [33] aimed to assess the efficacy of zolpidem (a positive modulator of GABAA receptors) in increasing the level of consciousness in three children with PVS by using [^18^F]FDG PET imaging to assess the effect of the therapy; however, no significant differences in regional cerebral metabolic activity were reported after treatment or after placebo.

Phillips et al. [34] proposed research aimed to disentangle VS/UWS patients from subjects affected by locked-in syndrome (LIS) (i.e., quadriplegic and mute but conscious patients) by an automatic procedure based on machine learning using [^18^F]FDG PET imaging. Their model had a 100% classification accuracy in distinguishing between the LIS and VS/UWS. Moreover, they reported that patients with LIS had a high probability of being similar to HC in terms of brain glucose metabolism.

Thibaut et al. [35] investigated brain glucose metabolism of extrinsic and intrinsic awareness networks. Metabolic dysfunction was demonstrated in patients with VS/UWS in both thalami and in a widespread cortical network encompassing the extrinsic/lateral network (i.e., bilateral posterior parietal and prefrontal areas) and the intrinsic/medial network (i.e., the precuneus and adjacent posterior cingulate cortex and mesiofrontal and adjacent anterior cingulate cortex), compared to HC. Additionally, progressive recovery of extrinsic and intrinsic awareness network activity, measured by [^18^F] FDG-PET imaging, was observed, ranging from VS/UWS to MCS and LIS.

Kim et al. [36] analyzed the brain metabolism in patients with VS/UWS after post-resuscitation hypoxic-ischemic brain injuries (HIBI). Compared with HC, patients showed significant hypometabolism in the bilateral precuneus, bilateral posterior cingulate gyri, bilateral middle occipital gyri, bilateral superior parietal gyri, right superior frontal gyrus, right angular gyrus, right supramarginal gyrus, both middle frontal gyri, and bilateral precentral gyri. In contrast, increased metabolism was observed in the bilateral insular cortices, bilateral cerebella, the right inferior frontal gyrus, and brainstem. Additionally, short-course patients within 7 months after injury had hypometabolism in the right precuneus, left middle occipital gyrus, right inferior occipital gyrus, bilateral middle frontal gyri, right superior frontal gyrus, and bilateral precentral gyri, while a significant increase was reported in the bilateral insular cortices, bilateral cerebellum, and brainstem. In the case of cardiac arrest-related HIBI, decreased glucose metabolism was present in the bilateral superior parietal gyri, bilateral precuneus, bilateral middle and inferior occipital gyri, right middle temporal gyrus, and left superior occipital gyrus, while the right insular cortex, both cerebella and the brainstem showed increased metabolism. In the case of VS/UWS after respiratory failure, decreased metabolism was reported in the bilateral middle occipital gyri, left precuneus, bilateral middle frontal gyri, right superior frontal gyri, and bilateral precentral gyri, while the bilateral cerebella, bilateral insular cortices, right inferior frontal gyrus, and brainstem showed significantly increased metabolism. Finally, the coma recovery scale-revised (CRS-R) score was positively correlated with brain metabolism in the bilateral fusiform and superior temporal gyri.

Stender et al. [37] proposed a quantitative analysis that can differentiate VS/UWS from MCS. Regarding global glucose metabolism, all patients had reduced metabolic rates compared to HC, but MCS patients had higher rates than those patients with VS/UWS. The regions with the most significant differences belonged to the primary and associative sensory (visual, auditory, and somatosensory) and motor areas bilaterally, and differences between MCS and VS/UWS patients were also present in a larger frontoparietal region that included the fronto-, temporo-, and occipito-parietal junctions (maximum difference map), but with less significance. The median cortical glucose metabolic rates averaged 42% of normal in patients with VS/UWS, 55% of normal in patients with MCS, and 53% of normal in emergence cases of MCSThe differences between VS/UWS and MCS were most pronounced in the frontoparietal cortex, at 42% and 60% of normal, respectively. In the brainstem and thalamus, metabolism declined equally in the two conditions. In addition, the likelihood of the diagnosis emerging from VS/UWS to MCS was at a threshold of metabolic rate of 44% of normal in the entire cortex and above 46% of normal within the maximum difference map. Based on cortical metabolism, receiver operating characteristic (ROC) analysis revealed that patients with MCS and VS/UWS could be differentiated with 82% accuracy. The same group investigated the ability of both [^18^F]FDG PET imaging and functional MRI (fMRI) to clinically detect VS/UWS and MCS and to predict the recovery of these patients. PET had a high sensitivity (93%) for the identification of patients with MCS and a high congruence (85%) with the CRS-R scale and was able to clearly detect VS/UWS. Sensitivity and congruence were lower for the fMRI. Additionally, PET imaging was able to predict the outcome of 74% of the subjects, which is higher than the rate of fMRI [38].

More recently, Steinhoff et al. [39] assessed the effects of music therapy on different areas of the brain of patients with UWS using [^18^F]FDG PET imaging. In patients who received continuous music therapy during the study weeks, tracer uptake in the frontal, hippocampal, and cerebellar regions was higher than that in patients who had discontinuous therapy, who, at the final evaluation, had a general glucose metabolism lower than the basal level.

Bodart et al. [40], using the same cohort as a previously reported study, performed interesting research with the purpose of cross-validating [^18^F]FDG PET and EEG response to transcranial magnetic stimulation (perturbational complexity index [PCI]) and identifying signs of consciousness in subjects with DOC. Patients with MCS had relative preservation of glucose metabolism in the internal or external fronto-parietal network, while conscious patients had hypometabolism limited to the cerebellum, brainstem, and motor areas. In the case of UWS subjects, preservation of metabolism was demonstrated only in the brainstem or cerebellum. Notably, PET imaging and PCI revealed preserved metabolic rates and high complexity levels in four patients who were behaviorally unresponsive.

More recently, Golkowski et al. [41] aimed to simultaneously use EEG, PET, and MRI to endorse diagnosis and prognosis in patients with DOC. In particular, EEG band power, fMRI connectivity, and glucose metabolism have been assessed as potential biomarkers of preserved consciousness or favorable outcomes. Glucose metabolism in the occipital lobe was significantly higher in patients with MCS than in patients with VS/UWS, while delta band power was a prognostic marker for favorable outcomes. In addition, PET values in the occipital cortex were significantly correlated with the CRS-R on the day of measurement.

Zhang et al. [42] used event-related potential (ERP) and [^18^F]FDG PET to study the neural correlates of different behavioral responses to transcranial direct current stimulation (tDCS) between patients in VS/UWS and MCS. Differences in regional uptake between these groups and HC were demonstrated in the left caudate, right caudate, left precentral, left mid-temporal gyrus, left Heschl gyrus, left rolandic operculum, right Heschl gyrus, right superior temporal gyrus, left insula, left midfrontal gyrus, and orbital part of the inferior frontal gyrus, with UWS participants showing lower uptake compared to MCS and MCS showing lower uptake compared to HC. In particular, the cerebral metabolic rate of glucose ratio in the HC was smaller than that in the VS/UWS in the left thalamus, left precuneus, left anterior cingulum, right anterior cingulum, right posterior cingulum, left superior frontal gyrus, and right superior frontal gyrus. Moreover, the same parameter was smaller for VS/UWS subjects than for MCS patients in the right posterior cingulum, left superior frontal gyrus, and right superior frontal gyrus. Therefore, the idea that residual brain metabolism in the stimulated area is necessary for a behavioral response to tDCS was underlined.

Hermann et al. [43] aimed to use both functional and anatomical imaging to demonstrate that the habituation of the auditory startle reflex (hASR) can distinguish MCS from VS/UWS. They demonstrated that the hASR correlated with cortical metabolism since the metabolic index was higher in patients with extinguishable startle response (ASR-EX) than in those with inextinguishable startle response (ASR-IN), with a higher metabolic index in MCS-EX than in MCS-IN; however, there were no significant differences between VS-EX and VS-IN. Moreover, voxel-wise analysis restricted to the VS/UWS population showed a higher metabolism in the posterior and anterior cingulate, premotor area, and anterior prefrontal cortex in VS-EX patients than in VS-IN patients.

Carrière et al. [44] performed a study to determine whether auditory localization could be considered a new sign of MCS, using a multimodal approach, revealing that VS/UWS patients without auditory localization (NO-LOCA) had decreased [^18^F]FDG metabolism in a large bilateral fronto-parieto-occipital network, in particular, in the right ventral posterior cingulate cortex, left premotor cortex, left angular gyrus, left sensorimotor associative regions, bilateral frontal eye fields, and thalamus. Subjects with auditory localization (LOCA) showed regional decreased metabolism (compared to HC), with the hotspots located in the ventral anterior and posterior cingulate cortex, left premotor cortex, right frontal eye fields, right angular gyrus, right visual secondary and associative areas, and right fusiform gyrus. However, a direct comparison between VS/UWS LOCA and NO-LOCA did not show any statistical differences. Additionally, no significant difference in brain metabolism was found between patients with VS/UWS LOCA and MCS. Compared to patients with MCS, and VS/UWS, NO-LOCA showed hypometabolism in the bilateral visual secondary cortex, right primary auditory cortex, right precuneus, right primary somatosensory cortex, bilateral primary motor cortex, right premotor cortex, and right visual associative cortex.

More recently, Sattin et al. [45] explored the integrity of neural structures that are part of the visual system in patients with DOC manifesting reflexive behavior (visual blink) and those manifesting cognitively and cortically mediated behavior (visual pursuit). A significant difference was found in [^18^F]FDG PET/CT images between the two groups, with a cluster localized in the right calcarine cortex (Brodmann area [BA] 17) and the neighboring right lingual gyrus cortex (BA 18/19). Additionally, considering both PET and MRI data, the main structural differences between the two groups were found in the retrochiasmatic areas, in particular in the optic radiation and visual cortex (V1) area of the right hemisphere. This area was statistically less functionally impaired in patients who could perform visual pursuit. The same authors performed a similar study to explore whether and how much the neurophysiological and neuroimaging measures could be predictive of the visual behavior of patients with DOC, developing a series of predictive models to evaluate if the visual fixation response can support the MCS diagnosis and its differentiation from VS/UWS [47]. The authors reported that the model encompassing MRI and flash Visual Evoked Potentials data in a dichotomous form, and the model including the same predictors along with [^18^F]FDG PET data of the cluster centered in V1 and the MRI data concerning the right optic radiation were more suitable for detecting visual pursuit than the other models encompassing different sets of predictors.

Hermann et al. [46] aimed to determine the validity of [^18^F]FDG PET metabolic index of the best-preserved hemisphere (MIBH) for DOC diagnosis in comparison with EEG-based classification, also analyzing the added value of a multimodal approach combining both modalities. Patients with VS/UWS had significantly lower MIBH than those with MCS. At the optimal MIBH cut-off of 3.07, corresponding to 54% of the healthy controls metabolism, the accuracy was 84%, positive predictive value was 85%, negative predictive value was 78%, sensitivity was 85%, and specificity was 78%. MIBH also correlated strongly with the CRS-R score. Additionally, these performances were not significantly different from those of EEG, and the multimodal assessment combining both of these modalities identified VS/UWS patients with neural correlates of consciousness and patients with 6-month recovery of command-following, outperforming each technique taken in isolation.

Curatola et al. [48] evaluated the combination of nerve growth factor (NGF) and transcranial direct current stimulation treatments to improve outcomes in children with VS/UWS after out-of-hospital cardiac arrest in a multimodality imaging setting. Baseline [^18^F]FDG PET showed a marked and global reduction in tracer uptake at the cortical, subcortical, and cerebellar levels, while after two cycles of treatment, a marked increase in tracer uptake in specific brain areas was reported in all three cases. A similar group published a paper exploring the effects of intranasal human recombinant NGF (hr-NGF) administration on the brain functions of three children with post-traumatic UWS [49]. A relevant improvement in functional [^18^F]FDG PET images and EEG with a concomitant amelioration of the patients’ clinical and neurological conditions was demonstrated

Ma et al. [50] aimed to investigate the therapeutic effects of trigeminal nerve stimulation (TNS) in patients with prolonged DOC. Compared with the baseline, metabolism in the right parahippocampal cortex, right precuneus, and bilateral middle cingulate cortex of the TNS group increased significantly after 2 weeks of treatment, while there was no significant increase in metabolism in the sham group 2 weeks after treatment. Additionally, the sham group presented widespread hypometabolism compared to the TNS group. A strong significant correlation between the standardized uptake value ratio (SUVR) parahippocampal and GCS changes from pre- to post-test was reported, showing that higher levels of brain metabolism improvement may be accompanied by stronger consciousness improvements. Despite these findings, it was specified whether VS/UWS subjects were part of the group that underwent PET evaluation.

More recently, Usami et al. [51] evaluated the cerebral glucose metabolism of patients with chronic DOC using [^18^F]FDG PET imaging. Cerebral metabolism in both the right and left hemispheres decreased with deteriorating levels of consciousness among patients. In particular, in the right hemisphere, the corpus callosum, central region, parietal region, angular gyrus, temporal region, occipital region, pericallosal region, lenticular nucleus, thalamus, hippocampus, and cerebellar hemisphere. In the left hemisphere, the corpus callosum, central region, parietal region, angular gyrus, temporal region, occipital region, pericallosal region, lenticular nucleus, thalamus, hippocampus, and cerebellar hemisphere were analyzed. Additionally, in the left hemisphere, regional glucose metabolism in the occipital region was significantly higher in the MCS group than in the VS/UWS group.

Guo et al. [52] explored the value of glucose metabolic pattern (GMP) obtained from [^18^F]FDG PET in distinguishing between UWS and MCS. Compared with HC, UWS GMP exhibited widespread hypometabolism in the bilateral frontoparietal cortex, including the inferior frontal gyrus, middle frontal gyrus, and inferior parietal lobule, coupled with hypermetabolism in the subcortical regions, including the midbrain, unilateral parahippocampal gyrus, lentiform nucleus, thalamus, anterior cingulate gyrus, and bilateral cerebellar hemispheres. Moreover, compared with MCS, UWS GMP was characterized by hypometabolism in the frontal–parietal cortex, including the precuneus, precentral gyrus, postcentral gyrus, paracentral lobule, frontal lobe, and parietal lobe, while hypermetabolism was observed in the lentiform nucleus, putamen, and anterior cingulate gyrus. The mean GMP expression score was significantly higher in UWS than in HC, and ROC analysis yielded an AUC of 0.975, with a cut-off value of 0.781. The mean GMP expression score was significantly higher in the UWS group than in the MCS group. The classification tasks comparing UWS and MCS exhibited an AUC of the GMP score of 0.77, with a cut-off value of 0.351 for distinguishing MCS from UWS. The GMP expression score had better performance in distinguishing between these clinical conditions than the cortical SUVR. Additionally, the UWS-MCS-GMP expression score and frontoparietal cortex SUVR were significantly correlated with the CRS-R score. Furthermore, the GMP expression score correctly predicted 73.7% of outcomes, with 72.2% sensitivity and 76.2% specificity in manifesting consciousness at follow-up, while the frontoparietal cortex SUVR correctly predicted 50.9% of outcomes, with the same sensitivity but only 14.3% specificity.

He et al. [53] aimed to establish a distinctive DOC-related pattern (DOCRP) on [^18^F]FDG PET imaging for assessing disease severity and distinguishing UWS from MCS. DOCRP was characterized bilaterally by relatively decreased metabolic activity in the medial and lateral frontal lobes, parieto-temporal lobes, cingulate gyrus, and caudate, associated with relatively increased metabolic activity in the cerebellum and brainstem, and had good performance in differentiating between HC and patients with DOC. Moreover, a significant difference in DOCRP expression between UWS and MCS patients was reported, with the first group having significantly higher scores, with an AUC value of 0.821 and an optimal cut-off value of 11.931, with a sensitivity of 85.7 % and specificity of 75.0 %. Notably, in the subgroup of patients with DOC caused by hypoxic-ischemic brain injury, DOCRP expression demonstrated even higher accuracy.

Lastly, Liu et al. [54] performed a study with the objective of employing [^18^F]FDG PET imaging to evaluate the resting-state brain metabolism in patients with DOC to identify objective quantitative metabolic indicators and predictors that could potentially indicate their level of awareness. A significantly different global cortical metabolic index between the MCS and UWS groups was demonstrated, with high AUCs in both the derivation and validation cohorts. A diagnostic accuracy of 0.89, as measured by the AUC across the combined cohort, was reported, with sensitivity and specificity rates of 88 and 81%, respectively. Focusing on regional metabolism, significant differences between the two groups in the left occipital lobe and the left precuneus were reported, with a lower metabolic rate in the UWS group. Additionally, differences in metabolic activity attributable to different etiologies were demonstrated since patients with TBI exhibit significant hypometabolism in the thalamus, with subsequent reductions in metabolism observed in the superior frontal gyrus, middle frontal gyrus, and angular gyrus, while subjects with cerebral hemorrhage exhibited notable hypometabolism in the posterior cingulate gyrus, external cerebellum, and thalamus. Conversely, patients with brainstem hemorrhage exhibited reduced metabolism in the cerebellum and middle frontal gyrus.

### 3.3. Studies with Mixed Tracers

First, Levy et al. [20] aimed to evaluate differences in cerebral blood flow (rCBF) and glucose metabolism in patients with VS/UWS and LIS using both [^18^F]FDG and [^15^O]CO_2_ PET. Patients in VS/UWS had a cortical gray metabolism reduced by more than 60% compared to HC, with the cerebellum showing a less evident reduction. In contrast, the LIS subjects showed only moderate metabolic depression. The regional cerebral glucose metabolism in patients with LIS was similar to that of HC, while it was blunted in patients with VS/UWS. Regional cerebral blood flow was depressed in VS/UWS subjects but not in the single LIS patient studied, and these parameters were reduced by nearly 50% of HC. In patients with VS/UWS, there was greater interpatient variability in rCBF than in regional cerebral glucose metabolism.

Laurey et al. [24] used both [^18^F]FDG and [^15^O]H_2_O PET imaging to investigate the auditory processing in VS/UWS subjects, revealing that resting metabolism of BA 41 and 22 were 61 and 64% lower in patients than in HC. During auditory stimulation, VS/UWS showed a significant increase in rCBF in both the transversal temporal gyri of Heschl (BA 41) and in the superior temporal gyrus on its superior surface (BA 42), as seen in HC, while differently to them the temporo-parietal superior temporal sulcus (BA 22/40) contralateral to the side of presentation failed to be activated. A significant alteration in the functional connectivity between BA 41, opposite to the side of auditory stimulation, and the controlateral BA 41 and 22, and the ispilateral posterior cingulate cortex and precuneus were demonstrated. Finally, BA 22, opposite the side of auditory stimulation, altered connectivity with the contralateral hippocampus, posterior parietal association cortex, and anterior cingulate cortex. The same group also published a study with the objective of measuring changes in rCBF during noxious stimulation compared with rest in persistent VS/UWS [25]. The mean metabolic rate in the overall gray matter in patients with PVS was less than 40% of that in HC. In HC, stimulation resulted in the subjective experience of pain and increased rCBF in the midbrain, contralateral thalamus, contralateral primary somatosensory, contralateral secondary somatosensory, bilateral insular, posterior parietal, and anterior cingulate cortices, while in PVS, stimulation increased regional neural activity in the brainstem and contralateral thalamus and primary somatosensory. Brain regions that showed significantly less activation in patients than in HC were identified in hierarchically higher-order cortices, including the contralateral secondary somatosensory cortex, bilateral insula, caudal and rostral anterior cingulate, and bilateral posterior parietal cortices. Differences in modulatory interactions from and to the primary somatosensory cortex in PVS compared those to in HC were demonstrated. In particular, the primary somatosensory cortex no longer experienced direct or indirect functional relationships with the secondary somatosensory, premotor, posterior parietal, superior temporal, and prefrontal cortices.

Rudolf et al. [26] assessed the correlation between glucose metabolism and benzodiazepine receptor density in the acute VS/UWS by using both [^18^F]FDG and [^11^C]flumazenil ([^11^C]FMZ) PET imaging. Global glucose metabolism in patients was below the lower threshold of normal values in all regions but without statistical significance. [^11^C]FMZ PET documented a reduction in global benzodiazepine binding sites in all cortical regions in VS/UWS, significantly different from HC in all of them, with the exception of the temporal cortex. In contrast, cerebellar [^11^C]FMZ binding in the VS/UWS group was nearly identical to that in the HC group. The reduction in glucose metabolism seemed to be in parallel with the reduction in cortical relative [^11^C]FMZ binding, with the only exception found in the cerebellum, where FMZ binding was normal, whereas metabolism was considerably reduced. A loose but significant correlation was observed between the two parameters. Patients who would eventually die could not be differentiated from patients who survived in a PVS based on PET findings performed with both tracers.

Lastly, Kassubek et al. [28] used both [^18^F]FDG and [^15^O]H_2_O PET imaging to evaluate the activation of a residual cortical network during painful stimulation in patients with long-term postanoxic VS/UWS. Significant perfusion changes in brain areas ipsilateral and contralateral to the stimulation were demonstrated, with significant hyperperfusion in the hemisphere opposite to the stimulus application in the posterior insula/secondary somatosensory, postcentral gyrus/primary somatosensory, and cingulate gyrus. In addition, a significant increase in perfusion was found in the posterior insula in the hemisphere ipsilateral to the stimulus. [^18^F]FDG PET highlighted widespread marked hypometabolism in the parietal and frontotemporal cortices, cuneus and precuneus, cingulum, frontal medial gyrus, precentral gyrus, bilateral thalamus, and transverse temporal gyrus, with a bihemispherical and nearly symmetrical pattern. The global maximum of regional hypometabolism was localized to the left inferior parietal cortex.

## 4. Discussion

Different papers have highlighted the possible role of [^18^F]FDG PET imaging in assessing altered brain glucose metabolism in patients with VS/UWS, providing useful information for evaluating the residual brain tissue function and assisting in the monitoring of brain recovery. VS/UWS is mainly associated with the presence of hypometabolic areas and alterations in cortical connectivity compared to HC, findings related to the presence of cortical disconnections. In general, VS/WS subjects had decreased glucose metabolism in the cortical and subcortical structures of the cerebral hemispheres and less diminished glucose metabolism in the brainstem. In particular, reduced metabolism was reported in the frontal lobe, temporal lobe, parietal lobe, and cingulum, while fewer reports underlined hypometabolism in the occipital lobe, thalamus, and cerebellum. Notably, hypermetabolism was also observed in the subcortical regions, vermis cerebelli, cerebellum, supramarginal cortex, insular cortices, right inferior frontal gyrus, and brainstem, as reported in other papers [21,24,26,27,30,31,32,35,36,52,55,56]. Moreover, focusing on symptom presentation, in patients with suspected blindness, visual cortex hypometabolism was present on the basis of altered visual evoked potential, while in subjects without fixation, metabolic dysfunction in a widespread cerebral network encompassing bilateral thalami and fronto-temporo-parietal associative cortices was demonstrated [21,56]. Additionally, some limited insights into the differences in areas involved in hypometabolism have been reported in some cases, depending on the etiology of VS/UWS or its duration [36,54]. Interestingly, some reports have proposed that patients in PVS have more reduced glucose metabolic activity than those in acute VS/UWS, with an exception reported for the frontal lobe, even though these findings were reported in different patients and not in a prospective setting with a follow-up of the same subjects [22,23]. Overall, [^18^F]FDG PET imaging seems to have the ability to assess brain metabolism in patients with VS/UWS and could be recommended for use in clinical practice, in particular in cases with challenging diagnoses and in the differentiation of other DOCs. Some insights into its ability to evaluate different DOC conditions have been reported, and future research could focus on the possibility of helping to define the level of consciousness of these patients. In this setting, it has been emphasized that the rate of misdiagnosis by bedside evaluation in the assessment of patients with DOCs is not negligible in some particular cases; therefore, [^18^F]FDG imaging could help reduce this rate [57]. This imaging modality might be particularly useful in supporting the clinical assessment of subtle signs of consciousness (such as visual fixation or eye tracking), thus facilitating a more accurate classification of DOC and helping to predict long-term outcomes. Moreover, the use of dynamic tests performed before and after a specific stimulus should also be promoted in the clinical assessment of these patients [58]. The combination of functional imaging and clinical assessment can aid clinical decision-making and rehabilitation planning. Furthermore, a better understanding of the underlying mechanisms of VS/UWS may contribute to optimizing therapeutic interventions. Moreover, the information obtained with [^18^F]FDG functional imaging may help understand the pathophysiological mechanisms underlying some of the symptoms that patients with VS/UWS present, along with the evolution of the condition over time.

Since the alterations in DOCs should be considered as a continuum, one of the most interesting insights underlined by the papers included in the review is the fact that the reduction in the level of consciousness seems to be related to worse findings on [^18^F]FDG PET imaging [42,51]. The gradual activation of the cortico-subcortical systems and subsequent activation of intercortical connectivity can help determine the most effective treatment for these patients. Interestingly, some studies have revealed a correlation between the CRS-R scale and PET results, even though these correlations have been reported in different brain regions in different papers [36,38,41,46,52]. Furthermore, several studies have investigated the ability of [^18^F]FDG PET imaging to differentiate VS/UWS from MCS based on the different distributions of hypometabolic areas, and in general, high sensitivity was reported [38]. The areas characterized by lower metabolic activity in VS/UWS subjects compared to MCS patients were the frontal lobe, parietal lobe, and occipital lobe, while less evidence was reported for the cingulum, caudate, brainstem, and thalamus. In addition, some reports have highlighted the presence of hypermetabolism in the cingulate, putamen, cerebellum, and brainstem. Interestingly, the relationship between electrical cerebral function and metabolism was impaired across regions of the brain in the VS/UWS group but was preserved in the MCS patients [25,26,28,31,37,41,46,51,52,53,54]. Similarly, a limited number of studies have revealed that patients with VS/UWS have lower metabolic activity than those with LIS [20,34]. Nevertheless, one study reported conflicting results, showing no differences in metabolic activity between MCS and VS/UWS patients [29]. These discrepancies could be due to several factors, such as the different methodologies used to assess tracer uptake (regional cerebral metabolic rate versus voxel-to-total brain uptake ratio), the inclusion of patients with different etiologies (brain trauma and hemorrhage), and the different procedures used to select the regions of interest. Interestingly, although Coleman et al. [29] did not underline differences in regional cerebral activity between MCS and VS/UWS, they reported that the coupling between this parameter and neuronal electrical activity was preserved in the first group and absent in the second group.

Some insights into the role of [^18^F]FDG PET imaging in assessing the prognosis of VS/UWS patients have also been reported, and in general, the preservation of hemispheric brain glucose metabolism seems to be indicative of the presence or potential recovery of consciousness [52]. In this setting, it has been proposed that a high baseline glucose metabolism may be a necessary if not sufficient, condition for the rise of conscious awareness by enabling long-distance connectivity across the cortex. Generally, the most severe patients showed the greatest reduction in their brain metabolism. In addition, though without a clear significance in the different studies, lower metabolism was observed in subjects with worse prognosis [23]. Interestingly, the combination of PET and EEG could predict the recovery of command-following, and PET prognostic ability was higher than fMRI in some reports [41,46].

As previously mentioned, fMRI could play a role in the evaluation of patients in VS/UWS since their functional assessment with this modality can help define their clinical status. The measurement of brain metabolism may be helpful in investigating brain functions that cannot be obtained by morphological and conventional imaging and can be used to assess the brain area responsible for consciousness. In this setting, some papers have directly or indirectly compared fMRI with [^18^F]FDG PET imaging, although with heterogeneous evidence. In particular, sensitivity and congruence for fMRI were lower than those for PET when identifying VS/UWS and MCS [38]. Moreover, the overlap between significant clusters of each imaging modality was relatively small, including the right operculum and left medial temporal gyrus, right caudate, and parts of the cingulum [30]. These findings need to be evaluated in a multimodality imaging setting, where different examinations can provide different and complementary information to assist in the clear and precise diagnosis of DOC, aiming for more accurate prognostications for these patients. As presented before, diagnostic tools, which include different modalities, are generally able to perform better than models based on single procedures.

[^18^F]FDG PET imaging can functionally and metabolically evaluate the brain, and different papers have investigated the ability of this imaging modality to assess the response of patients with VS/UWS to particular stimulation. In general, it has been proposed that residual brain activity in the target area should be present to determine a specific behavioral response. In particular, when assessing the response to tDCS, differences in cerebral tracer uptake in UWS/VS, MCS, and HC have been reported [42]. Similarly, significant alterations in the functional connectivity between VS/UWS patients and HC were also underlined after auditory stimulation, and differences in brain metabolism were observed between UWS patients with and without auditory localization [24,44]. Additionally, different findings on [^18^F]FDG imaging were observed between patients with DOC manifesting reflexive behavior (visual blink) and those manifesting cognitively and cortically mediated behavior (visual pursuit) [45]. Moreover, it has been reported that there is congruence between the preservation of tracer uptake in the brainstem or the cerebellum and PCI transcranial magnetic stimulation and that differences in hypometabolic patterns were present between inextinguishable and extinguishable startle responses with habituation of the auditory startle reflex [40,43]. PET imaging can also define an improvement in brain metabolism in the frontal, hippocampal, and cerebellar regions after music therapy [40]. Metabolic cerebral responses to pain provide deeper insights into the patients’ central nociceptive system, even though they can only complement the clinical assessment. Lastly, single and specific papers demonstrated the ability of [^18^F]FDG PET imaging to underline changes in brain metabolism related to NGF therapy, transcranial direct current stimulations, or TNS [48,49,50].

### 4.1. Limitations

Some limitations, mainly derived from the characteristics of the included papers, affect this review and our findings. Their previously described quality assessment clearly highlights this fact. First, most studies are characterized by small and heterogeneous cohorts, and in particular, in some of them, only a limited number of patients were in VS/UWS. Small sample sizes (e.g., Snyman et al. [33] and Curatola et al. [48]) limit the statistical power and generalizability of the results. Consequently, the descriptive analyses and conclusions drawn were based on limited and heterogeneous samples. Additionally, some studies included in the present review had a retrospective design.

The included studies exhibited significant variability in PET protocols; this significant heterogeneity, including variations in tracers (e.g., mixed use of [^18^F]FDG, [^15^O]H_2_O, and [^11^C]flumazenil), injected activities (when specified), devices (standalone PET, PET/CT, PET/MR), and analysis methods (e.g., visual versus quantitative metrics), directly impacted the validity and generalizability of the review’s conclusions. In particular, differences in scanner resolution (e.g., PET versus PET/CT) affect spatial accuracy, potentially affecting regional metabolic assessments (e.g., thalamic versus cortical hypometabolism). Variability in the injected activities influences image quality and quantitative metrics (e.g., SUVR), complicating cross-study comparisons of metabolic thresholds. Mixed tracers introduce confounding variables, and for example, [^15^O]H_2_O measures blood flow, not metabolism, while [^11^C]flumazenil targets benzodiazepine receptors, factors not accounted for in pooled analyses of “hypometabolism.” Studies using outdated PET scanners or non-standardized reconstructions (e.g., filtered back projection versus iterative methods) may underestimate glucose metabolism due to poorer noise reduction. The lack of harmonized patient preparation (e.g., fasting duration, ambient noise control) could alter baseline brain activity, particularly in patients with DOC with fluctuating arousal. Small, heterogeneous cohorts combined with protocol variability reduce the reliability of pooled findings. To mitigate these issues, the adoption of the European Association of Nuclear Medicine (EANM) procedural standards for brain [18F] FDG PET imaging is desirable [7]. In particular, efforts should be made to tend to technical harmonization: scanner calibration and reconstruction algorithms (e.g., ordered subset expectation maximization [OSEM] method with resolution recovery) to ensure consistent quantification; fixed injected activities (e.g., 3–5 MBq/kg) and uptake periods (30–40 min post-injection) to reduce inter-study variability; patient preparation with standardized fasting, quiet resting conditions, and avoidance of stimulants (e.g., caffeine) to minimize confounding metabolic fluctuations; data reporting: mandatory disclosure of scanner type, reconstruction parameters, and normalization methods (e.g., global mean versus cerebellar reference) to enable cross-validation.

Finally, there is a lack of clinical implementation guidance. Our systematic review synthesizes evidence on the diagnostic and prognostic utility of [^18^F] FDG PET in VS/UWS but fails to translate these findings into actionable clinical guidelines. For instance, while PET seems to be helpful in distinguishing VS/UWS from MCS via frontoparietal hypometabolism, clinicians are unclear on how to integrate this modality into practice. Despite behavioral assessments like the CRS-R remaining the gold standard for the evaluation of DOC patients relies on observable motor or verbal responses, which may be absent or inconsistent in patients with severe motor impairments or fluctuating arousals. Therefore, it suffers from subjectivity and is prone to inter-rater variability. PET imaging may help to resolve ambiguities in cases where the clinical scenario, together with the CRS-R evaluation, is inconclusive (e.g., covert awareness, atypical behaviors) by measuring cerebral glucose metabolism and identifying residual brain activity in regions associated with consciousness (e.g., frontoparietal networks, precuneus), even when behavioral signs are absent, providing quantitative and objective metrics (e.g., metabolic rates, connectivity patterns). This helps diagnose covert awareness in patients who are behaviorally unresponsive but retain neural correlates of consciousness. However, from the available evidence reported in this review, it must be confirmed that [^18^F]FDG PET can only complement clinical evaluation and not replace clinical judgment or behavioral assessments. For instance, a patient with frontoparietal hypometabolism but intermittent visual tracking on CRS-R warrants continued observation, even if PET suggests severe dysfunction. Furthermore, it is important to underline that metabolic activity and consciousness are not strictly correlated, are not tied together, and their relationship needs a deeper comprehension. Hypermetabolism in non-associative regions (e.g., the brainstem) may not correlate with meaningful recovery.

Most of the papers included in the present review did not present a quantitative evaluation of their findings, in agreement with the evaluation of cerebral [^18^F]FDG images, which is usually performed with a qualitative assessment. In addition, the papers focused on different fields of application of this imaging modality without a general specific topic. Therefore, no meta-analysis of the retrieved data could be performed.

As a consequence, even though our findings provide some insights into the role of [^18^F]FDG PET imaging in the evaluation of VS/UWS in different clinical settings, new efforts, larger prospective studies, and multicenter collaborations are required to clearly assess, confirm, or challenge its value, thereby strengthening the reliability of the review.

### 4.2. Ethical Implications

The clinical management of patients with DOC is strongly characterized by ethical issues [59]. As previously highlighted, PET findings could help in different clinical settings in the evaluation of patients with VS/UWS. In clinical decision-making, the interpretation of data resulting from this imaging modality carries profound ethical implications, particularly in emotionally charged scenarios such as withdrawal-of-care discussions. Moreover, the quality of life of patients with DOC should be a central point in their management and a fundamental outcome to pursue [60]. The quality of life of patients regaining some grades of consciousness is another pivotal point in the ethical discussion, and the possible implications of PET findings in this setting should be investigated in future studies. While PET imaging can reveal residual metabolic activity (e.g., cerebellar hypermetabolism [36]), these findings must be interpreted cautiously to avoid misleading families or clinicians. As a matter of fact, we underlined some insights into the role of PET imaging in the prediction of outcomes, which may help in the definition of recovery from VS/UWS [61]. However, several issues and findings require further exploration. For example, isolated cerebellar hypermetabolism might reflect preserved brainstem reflexes rather than conscious awareness, potentially fostering unrealistic hopes for recovery despite a patient’s globally poor prognosis. Conversely, widespread cortical hypometabolism could prematurely sway families toward withdrawing care, even in cases where subtle signs of consciousness are present. In regions where withdrawal decisions hinge on the “irreversible” loss of function, PET evidence of residual metabolism could conflict with the legal definitions of brain death or VS/UWS.

Cost-effectiveness and resource allocation are other pivotal issues that should be considered. Even though in different clinical settings and pathologies [^18^F]FDG PET imaging has proven its value, this imaging modality is costly and difficult to access in low-resource settings. Identifying the scenarios in which PET adds maximal value (e.g., prognosis prediction in traumatic versus anoxic brain injury and evaluation of response to therapy) would be desirable.

## 5. Conclusions

This review provides evidence for the possible role of [^18^F]FDG PET imaging in the assessment of patients with VS/UWS, particularly in differential diagnosis and prognosis. This imaging modality can be used to evaluate DOC, and its ability to differentiate between VS/UWS and other conditions, in particular MCS, has been reported. Moreover, some insights into its value in different clinical settings, such as stimulation response and therapy evaluation, have been proposed. However, standardization of protocols and larger prospective studies are needed to strengthen these clinical recommendations. Without clear clinical implementation strategies, [^18^F]FDG PET risks may remain a research tool. By proposing a clinical algorithm, addressing cost and access barriers, and integrating ethical frameworks, it is possible to bridge the gap between metabolic insights and bedside decision-making, ultimately improving care for patients with DOC.

## Figures and Tables

**Table 1:** Characteristics of the studies included in the review.

Authors	N. Ref	Year	Country	Study Design	N. Pts.	VS/UWS (%)	Aetiology
Levy D.E. et al.	[20]	1987	USA, Canada	Prospective	10	7 (70)	CPA, trauma, anesthesia-hypoxia and asphyxia
De Volder A.G. et al.	[21]	1994	Belgium	Prospective	12	7 (58)	CPA
Tommasino C. et al.	[22]	1995	Italy	Prospective	25	10 (40)	Cerebrovascular accident, trauma and CPA
Rudolf J. et al.	[23]	1999	Germany	Prospective	24	24 (100)	CPA, primary ipo-anoxia due to myasthenic crisis, attempted suicide, anesthesia
Laureys S. et al.	[55]	1999	Belgium	Retrospective	82	4 (5)	intoxication, CPA, trauma
Laureys S. et al.	[24]	2000	Belgium	Prospective	58	5 (9)	CPA
Laureys S. et al.	[25]	2002	Belgium	Prospective	30	15 (50)	CPA, anoxia, drug abuse, encephalitis, and intoxication
Rudolf J. et al.	[26]	2002	Germany	Prospective	18	9 (50)	CPA
Beuthien-Baumann B. et al.	[27]	2003	Germany	Prospective	23	16 (69)	Trauma, hemorrhage
Kassubek J. et al.	[28]	2003	Germany	Prospective	7	7 (100)	CPA, intoxication,
Coleman M.R. et al.	[29]	2005	UK	Prospective	10	6 (60)	Trauma, hemorrhage
Juengling F.D. et al.	[30]	2005	Switzerland, Germany	Prospective	5	5 (100)	CPA, intoxication
Nakayama N. et al.	[31]	2006	Japan	Retrospective	82	17 (21)	Trauma
Bruno M.A. et al.	[56]	2010	Belgium	Prospective	49	10 (20)	Anoxia
Kim Y.W. et al.	[32]	2010	Republic of Korea	Prospective	24	12 (50)	Trauma, hypoxia, cerebrovascular injury
Snyman N. et al.	[33]	2010	Australia	Prospective	3	3 (100)	Trauma, drowning
Phillips C.L. et al.	[34]	2011	Belgium	Retrospective	82	13 (16)	Anoxia, CPA, trauma, thrombosis, hemorrhage
Thibaut A. et al.	[35]	2012	Belgium	Prospective	70	24 (34)	CPA, anoxia, trauma, stroke, meningitis, hemorrhage
Kim Y.W. et al.	[36]	2013	Republic of Korea	Prospective	32	17 (53)	CPA
Stender J. et al.	[37]	2014	Denmark, Belgium, Australia, USA, Canada	Retrospective	41	14 (34)	Trauma, CPA, infection, stroke, hemorrhage
Stender J. et al.	[38]	2014	Belgium, Canada, Denmark	Retrospective	12	41 (33)	Trauma, cardiac arrest, stroke, hemorrhage, infection and metabolic disorders
Steinhoff N. et al.	[39]	2015	Austria	Prospective	4	4 (100)	ns
Bodart O. et al.	[40]	2017	Belgium, USA, Italy, Brazil	Retrospective	24	9 (38)	Trauma and non-traumatic injuries
Golkowski D. et al.	[41]	2017	Germany	Prospective	20	14 (70)	Trauma, stroke, anoxia, metabolic
Zhang Y. et al.	[42]	2019	China	Prospective	19	8 (42)	Anoxia, trauma, hemorrhage
Hermann B. et al.	[43]	2020	France, Belgium, USA	Prospective	96	48 (50)	Anoxia, trauma, hemorrhage
Carrière M. et al.	[44]	2020	Belgium, UK	Retrospective	186	64 (34)	Trauma and non-traumatic injuries
Sattin D. et al.	[45]	2020	Italy	Retrospective	54	42 (78)	Trauma, anoxia, ischemia and hemorrhage
Hermann B. et al.	[46]	2021	France, Belgium	Prospective	52	21 (40)	Trauma, anoxia, ischemia and hemorrhage, intoxication, encephalomielitis
Sattin D. et al.	[47]	2021	Italy	Retrospective	58	42 (72)	Trauma, anoxia, ischemia and hemorrhage
Curatola A. et al.	[48]	2023	Italy	Prospective	3	3 (100)	PCA
Gatto A. et al.	[49]	2023	Italy	Prospective	3	3 (100)	Trauma
Ma H. et al.	[50]	2023	China	Prospective	60	53 (88)	Anoxia, trauma, and stroke
Usami N. et al.	[51]	2023	Japan	Retrospective	50	15 (30)	Trauma
Guo K. et al.	[52]	2024	China	Prospective	57	21 (37)	Trauma, hemorrhage, ischemia, toxic encephalopathy.
He Z. et al.	[53]	2024	China	Prospective	15	7 (47)	Trauma, hypoxia, ischemia
Liu D. et al.	[54]	2025	China, UK	Retrospective	56	21 (38)	Trauma, hypoxia–ischemia, hemorrhage

N.: number; Pts: patients; Ref.: reference; VS/UWS: vegetative state/unresponsive wakefulness syndrome; USA: United States of America; UK: United Kingdom; CPA: cardiopulmonary arrest.

**Table 2:** Results and main findings of the studies considered for review.

Authors	Ref.	Device and Tracers	Reported Mean Activity (MBq)	DOCs Comparison	Cerebral Regions	Main Findings
Levy D.E.et al.	[20]	PET; [^15^O]CO_2_, [^18^F]FDG	555/min for 8 min for [^15^O]CO_2_, 185–370 for [^18^F]FDG	VS vs. LIS	All	Cortical glucose metabolism is more reduced in VS than in LIS patients. In VS, this reduction is present also in basal nuclei and cerebellum. Cerebral blood flow exhibits more variable reduction.
DeVolder A.G. et al.	[21]	PET; [^18^F]FDG	ns	na	Parieto-occipital cortex, frontomesial junction, striatum, and visual cortex	Cerebral glucose metabolism is decreased in VS. The most consistent regional alterations are in the parieto-occipital cortex.
Tommasino C. et al.	[22]	PET; [^18^F]FDG	3.7/Kg	VS vs. coma, VS vs. PVS	All	Coma and VS patients have reduced brain glucose metabolism with overlapping patterns. Higher metabolic activity is found in the primary visual cortex of VS subjects compared to coma patients. PVS subjects have a more reduced metabolism compared to VS.
Rudolf J. et al.	[23]	PET; [^18^F]FDG	370	PVS vs. AVS	All	Glucose metabolism is reduced in VS patients, with a significantly lower metabolism in PVS compared to AVS.
Laureys S. et al.	[55]	PET; [^18^F]FDG	185–370	na	Prefrontal, premotor, and parietotemporal association areas and posterior cingulate cortex/precuneus	Impaired glucose metabolism in the prefrontal, premotor, and parietotemporal association areas and posterior cingulate cortex/precuneus is present in VS. Various prefrontal and premotor areas are less tightly connected with the posterior cingulate cortex than in HC.
Laureys S. et al.	[24]	PET; [^18^F]FDG and [^15^O]H_2_O	185–370 for [^18^F]FDG, 222–296 for [^15^O]H_2_O	na	The study focuses on Brodmann areas 41 and 42	VS patients have lower resting metabolism but maintain the activation of bilateral auditory areas after stimulation. Temporo-parietal junction fails to activate.
Laureys S. et al.	[25]	PET; [^18^F]FDG and [^15^O]H_2_O	185–370 for [^18^F]FDG, 222 for [^15^O]H_2_O	na	All	VS patients have reduced brain glucose metabolism. However, somatosensory stimulation activates the midbrain, contralateral thalamus, and primary somatosensory cortex, even in the absence of detectable cortical activation. Secondary somatosensory, bilateral insular, posterior parietal, and anterior cingulate cortices did not show activation. The activated primary somatosensory cortex is functionally disconnected from secondary somatosensory, bilateral posterior parietal, premotor, polysensory superior temporal, and prefrontal cortices.
Rudolf J. et al.	[26]	PET; [^18^F]FDG and [^11^C]flumazenil	Ns for [^18^F]FDG, 740 for [^11^C]flumazenil	na	All supratentorial cortical regions	VS patients have overall reduced glucose metabolism. Benzodiazepine receptor binding sites are also reduced, with a gross correspondence between the two alterations.
Beuthien-Baumann B. et al.	[27]	PET; [^18^F]FDG	300	na	Frontal cortex, temporal cortex, less frequently occipital cortex, parietal cortex, and cerebellum	VS patients demonstrate a reduction in glucose metabolism, with less grade in the vermis cerebelli, similarl to the reduction in perfusion at SPECT images.
Kassubek J. et al.	[28]	PET; [^15^O]H_2_O and [^18^F]FDG	1000 for [^15^O]H_2_O, 200 ± 20 for [^18^F]FDG	na	Parietal and frontotemporal cortices, cuneus and precuneus, cingulum, frontal medial gyrus, the precentral gyrus, transverse temporal gyrus and thalamus,	VS subjects have widespread hypometabolism. Pain-induced hyperperfusion is present in the posterior insula/secondary somatosensory cortex, postcentral gyrus/primary somatosensory cortex, the cingulate cortex contralateral to the stimulus, and in the posterior insula ipsilateral to the stimulus.
Coleman M.R. et al.	[29]	PET; [^18^F]FDG	74	MCS vs. VS	All	The coupling between neuronal electrical activity and regional glucose metabolism is preserved in MCS patients but absent in VS subjects.
Juengling F.D. et al.	[30]	PET; [^18^F]FDG	200 ± 20	na	Parietal, parietooccipital, and frontotemporal cortices, cingulum, frontal medial and precentral gyrus, and bilateral thalamus.	VS subjects demonstrate widespread hypometabolism in the parietal, parietooccipital, and frontotemporal cortices, cingulum, frontal medial and precentral gyrus, and within the bilateral thalamus.
Nakayama N. et al.	[31]	PET; [^18^F]FDG	4.44/Kg	VS vs. MCS	Medial prefrontal regions, the medial frontobasal regions, the cingulate gyrus, and the thalamus.	Bilateral hypometabolism in the medial prefrontal regions, the medial frontobasal regions, the cingulate gyrus, and the thalamus is most widespread and prominent in VS patients compared to MCS and higher brain dysfunction subjects.
Bruno M.A. et al.	[56]	PET; [^18^F]FDG	ns	na	Fronto-parietal cortical network	Patients without fixation show metabolic dysfunction in fronto-parietal cortical network, which is not different from the brain function in patients with visual fixation.
Kim Y.W. et al.	[32]	PET; [^18^F]FDG	555	na	Left precuneus, both posterior cingulate cortices, left superior parietal lobule, cerebellum, and right supramarginal cortex.	Patients in PVS have decreased glucose metabolism in the left precuneus, both posterior cingulate cortices, and the left superior parietal lobule and increased metabolism in both the cerebellum and the right supramarginal cortices. Decreased level of consciousness is correlated with decreased metabolism in both posterior cingulate cortices.
Snyman N.. et al.	[33]	PET/CT; [^18^F]FDG	370 scaled to weight	na	na	No objective changes in PET after zolpidem therapy are demonstrated in pediatric PVS patients.
Phillips C.L et al.	[34]	PET; [^18^F]FDG	185–370	VS vs. LIS	Brainstem, bilateral lower temporal lobe and cerebellum	A classifier able to distinguish LIS and VS with good performances is built.
Thibaut A. et al.	[35]	PET; [^18^F]FDG	185–370	VS vs. MCS, VS vs. LIS	Thalami and cortical network encompassing the extrinsic/lateral network and the intrinsic/medial network.	VS/UWS patients show metabolic dysfunction in extrinsic and intrinsic networks and thalami.
Kim Y.W. et al.	[36]	PET; [^18^F]FDG	555	na	Decreased metabolism in bilateral precuneus, bilateral posterior cingulate gyrus, bilateral middle frontal gyri, bilateral superior parietal gyri, bilateral middle occipital gyri, bilateral precentral gyri, and increased metabolism in bilateral insula, bilateral cerebella, and the brainstem	VS demonstrates decreased metabolism in bilateral precuneus, bilateral posterior cingulate gyrus, bilateral middle frontal gyri, bilateral superior parietal gyri, bilateral middle occipital gyri, bilateral precentral gyri, and increased brain metabolism in bilateral insula, bilateral cerebella, and the brainstem. Level of consciousness is correlated with brain metabolism in bilateral fusiform and superior temporal gyri.
Stender J. et al.	[37]	PET/CT; [^18^F]FDG	185–370	VS vs. MCS	Frontoparietal cortex, brainstem, and thalamus	Differences in glucose metabolism between VS/UWS and MCS are most pronounced in the frontoparietal cortex. In the brainstem and thalamus, metabolism declines equally.
Stender J. et al.	[38]	ns; [^18^F]FDG	ns	na	ns	PET could be used to complement bedside examinations and predict the long-term recovery of patients with UWS. MRI seems to be less accurate.
Steinhoff N. et al.	[39]	PET/CT; [^18^F]FDG	230	na	Frontal, hippocampal, and cerebellar	Tracer uptake in frontal, hippocampal, and cerebellar regions is higher in VS patients after music therapy.
Bodart O. et al.	[40]	PET/CT; [^18^F]FDG	185–370	na	Preservation of the metabolism only in the brainstem or the cerebellum	PET and EEG provide congruent results, revealing preserved metabolic rates and high complexity levels in patients who are behaviourally unresponsive.
Golkowski D. et al.	[41]	PET/MR; [^18^F]FDG	185	VS vs. MCS	Occipital cortex	Glucose metabolism in the occipital lobe is significantly higher in MCS than in VS patients.
Zhang Y. et al.	[42]	PET/CT; [^18^F]FDG	3.7–7.4 Kg	VS vs. MCS	Left caudate, right caudate, left precentral, left mid-temporal gyrus, left Heschl gyrus, left rolandic operculum, right Heschl gyrus, right superior temporal gyrus, left insula, left midfrontal gyrus and orbital part of inferior frontal gyrus	Reduced metabolism is present in VS patients compared to MCS and HC after transcranial direct current stimulation. A residual brain activity in stimulated areas is necessary for behavioral response to transcranial direct current stimulation.
Hermann B. et al.	[43]	PET; [^18^F]FDG	ns	VS vs. MCS	Posterior and anterior cingulate, premotor area and anterior prefrontal cortex	The presence of auditory startle reflex is suggestive of an MCS or conscious state and correlates with the functional preservation of large-scale cortical networks related to conscious processing and voluntary inhibitory control of behaviour.
Carrière M. et al.	[44]	PET; [^18^F]FDG	185–370	VS vs. MCS	fronto-parieto-occipital	Patients without auditory localization show decreased metabolism in a large bilateral fronto-parieto-occipital network. Patients with localization show regional decreased metabolism with the hotspots located in the ventral anterior and posterior cingulate cortex left premotor cortex, right frontal eye fields, right angular gyrus, right visual secondary and associative areas, and right fusiform gyrus.
Sattin D. et al.	[45]	PET; [^18^F]FDG	140 ± 30	na	Right calcarine cortex and neighboring right lingual gyrus.	PET underlines differences in the right calcarine cortex and neighboring right lingual gyrus between patients with reflexive behavior and those manifesting a cognitively and cortically mediated behavior after visual stimuli.
Hermann B. et al.	[46]	PET/CT; [^18^F]FDG	2/Kg	VS vs. MCS	Best preserved hemisphere	[^18^F]FDG metabolic index of the best-preserved hemisphere has good diagnostic performances in distinguishing between MCS and VS subjects.
Sattin D. et al.	[47]	PET; [^18^F]FDG	140 ± 30	VS vs. MCS	na	PET data could be included in a model with good performances to predict visual fixation to support differential diagnosis in DOC.
Curatola A. et al.	[48]	PET; [^18^F]FDG	3/Kg	na	All	The improvement derived from treatment with NGF and transcranial direct current stimulations can be assessed with PET imaging.
Gatto A. et al.	[49]	PET; [^18^F]FDG	3/Kg	na	All	The improvement of treatment with intranasal human-recombinant NGF can be assessed with PET imaging.
Ma H. et al.	[50]	PET/CT; [^18^F]FDG	150	na	Right parahippocampal cortex, right precuneus, and bilateral middle cingulate cortex	Glucose metabolism of the right parahippocampal cortex, right precuneus, and bilateral middle cingulate cortex are significantly increased in DOC patients treated with trigeminal nerve stimulation.
Usami N. et al.	[51]	PET; [^18^F]FDG	3.5/Kg	VS vs. MCS	Left occipital region	Glucose metabolism reduces with consciousness deterioration in cerebral regions. Metabolic activity is higher in the left occipital region in MCS—patients than in VS subjects.
Guo K. et al.	[52]	PET/CT; [^18^F]FDG	3.7/Kg	VS vs. MCS	Hypometabolism in the frontal–parietal cortex, hypermetabolism in the unilateral lentiform nucleus, putamen, and ante- rior cingulate gyrus	A PET-based score was able to distinguish between UWS and MCS with good performances and to predict the outcome is built.
He Z. et al.	[53]	PET/CT; [^18^F]FDG	185	VS vs. MCS	Hypometabolism in the medial and lateral frontal lobes, parieto-temporal lobes, cingulate gyrus, and caudate increased metabolism in the cerebellum and brainstem	A DOC-related pattern of decreased metabolism is present in the medial and lateral frontal lobes, parieto-temporal lobes, cingulate gyrus, and caudate, associated with relatively increased metabolism in the cerebellum and brainstem. This pattern has good accuracy in differentiating between MCS and UWS.
Liu D. et al.	[54]	PET/CT; [^18^F]FDG	3.7/Kg	VS vs. MCS	Left occipital lobe and the left precuneus	There is a significant difference in cortical metabolism index between MCS and UWS.

CO_2_: carbon dioxide; [^18^F]FDG: ^18^F-fluorodeoxyglucose; VS: vegetative state; ns: not specified; Kg: kilogram; PVS: persistent VS; AVS: acute VS; PET: positron emission tomography; CT: computed tomography; H_2_O: water; MCS: minimally conscious state; MBq: megabecquerel; Ref: reference; MRI: magnetic resonance imaging; EEG: electroencephalogram; NGF: nerve growth factor; UWS: unresponsive wakefulness syndrome; DOC: disorders of consciousness; LIS: locked-in syndrome; HC: healthy controls; ns: not specified; na: a comparison between DOCs has not been performed or hypometabolism in specific regions has not been reported.

## Data Availability

Data supporting the reported results can be found in the PubMed/MEDLINE, Scopus, and Embase databases.

## Supplementary Materials

The following supporting information can be downloaded at: https://www.mdpi.com/article/10.3390/diagnostics15111406/s1, PRISMA 2020 Checklist.
